# Supplementary material for: KK-LC-1 as a therapeutic target to eliminate ALDH+ stem cells in triple negative breast cancer
Source: Nat Commun. 2023 May 5;14:2602. doi: 10.1038/s41467-023-38097-1 (PMC10163259; doi:10.1038/s41467-023-38097-1)
Supplement: Supplementary file 1 — Supplementary Information [file 41467_2023_38097_MOESM1_ESM.pdf]

Supplementary Information

KK-LC-1 as a therapeutic target to eliminate ALDH+ stem cells in triple negative breast cancer

Supplementary Fig. 1

Supplementary Fig. 2

Supplementary Fig. 3

Supplementary Fig. 4

Supplementary Fig. 5

Supplementary Fig. 6

Supplementary Fig. 7

Supplementary Fig. 8

Supplementary Fig. 9

Supplementary Fig. 10

Supplementary Fig. 11

Supplementary Table 1

Supplementary Table 2

Supplementary Table 3

Supplementary Table 4

Supplementary Table 5

Supplementary Table 6

Supplementary Fig 1

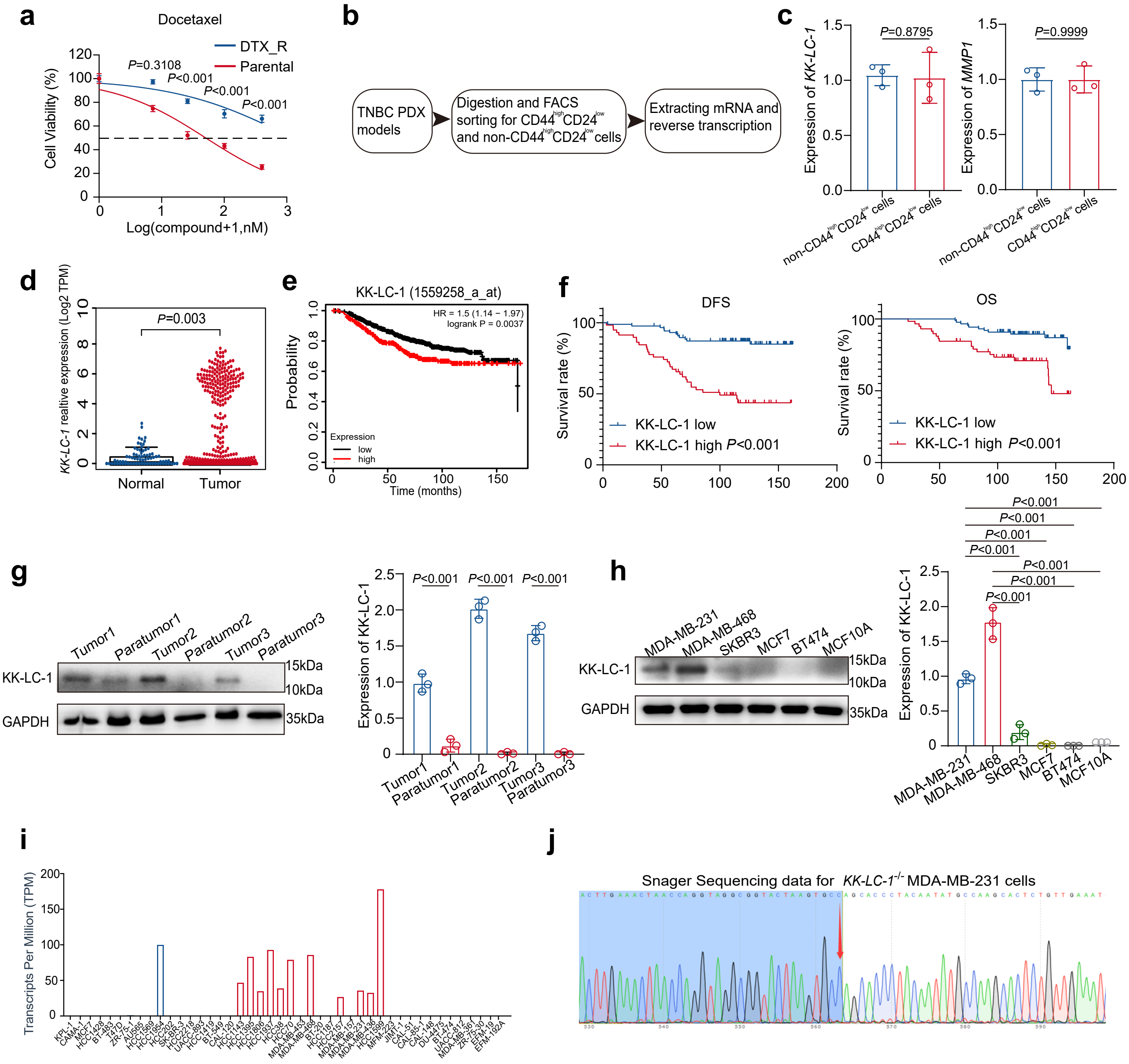

Supplementary Fig. 1

KK-LC-1 is highly expressed in TNBC cancerous tissue and associated with poor prognosis.

a: Drug susceptibility test of docetaxel-resistant MDA-MB-231 cells and parental MDA-MB-231 cells to docetaxel; data are presented as mean±SEM from three biologically independent experiments, statistical significance was determined using Student's test and was two sided.

b: Schematic showing the identification and isolation of primary $CD44^{high}CD24^{low}$ and non- $CD44^{high}CD24^{low}$ cells.

c: *KK-LC-1* and *MMP1* showed no significant change in mRNA expression in primary $CD44^{high}CD24^{low}$ and non-$CD44^{high}CD24^{low}$ cells; data are presented as mean±SD from three biologically independent experiments, statistical significance was determined using Student's t test and was two sided.

d: Expression profile of *KK-LC-1* mRNA in tumor and normal tissues in the TCGA dataset, statistical significance was determined using Student's t test and was two sided.

e: Survival analysis of patients with high or low *KK-LC-1* expression in the KM plotter database, statistical significance was determined using log rank test.

f: Disease-free survival analysis and overall survival analysis of patients with high or low KK-LC-1 expression in 144 TNBC patients, statistical significance was determined using log rank test.

g: Western blotting showing KK-LC-1 protein levels in TNBC tumor and para-tumor tissue samples; data are presented as mean±SD from three biologically independent experiments, statistical significance was determined using Student's t test and was two sided.

h: Western blotting showing KK-LC-1 protein levels in the MDA-MB-231, MDA-MB-468, SKBR3, MCF7, BT474 and MCF10A cell lines; data are presented as mean±SD from three biologically independent experiments, statistical significance was determined using Student's t test and was two sided.

i: Expression of *KK-LC-1* mRNA in multiple breast cancer cell lines.

j: Sanger sequencing data for *KK-LC-1*$^{-/-}$ MDA-MB-231 cells.

Source data are provided in the Source Data file.

Supplementary Fig 2

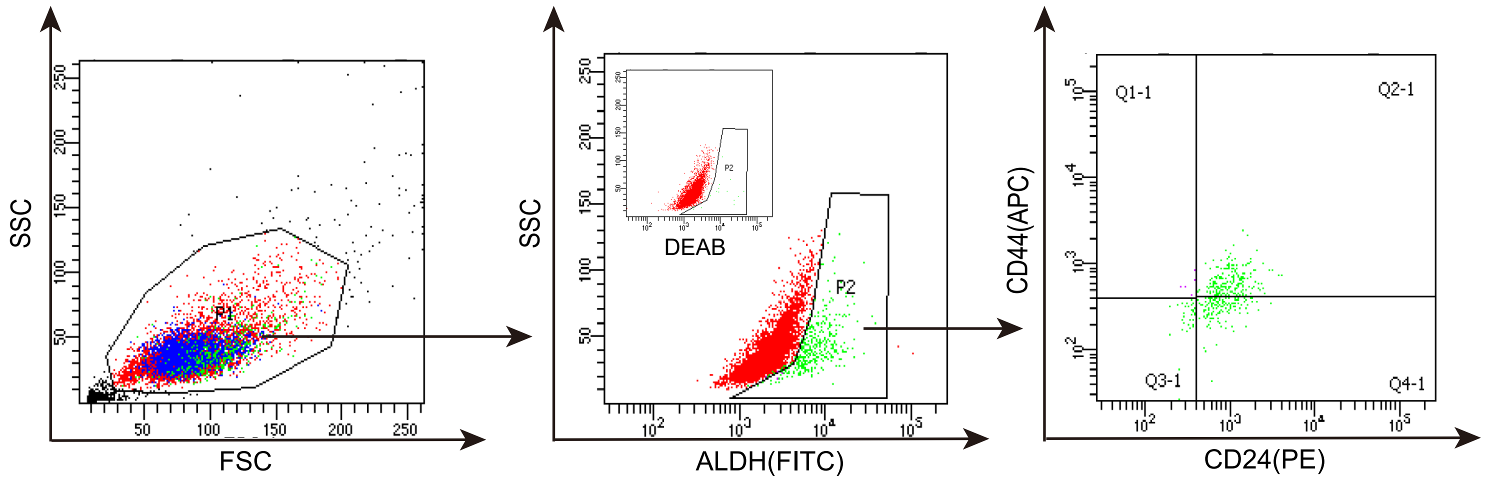

Supplementary Fig 2

Figure exemplifying the gating strategy in flow cytometry.

Gating strategy: cells were first gated using FSC and SSC, the dead cells which separated from the major population was removed in this step. Further on, cells from P1 were tested for ALDH activity. The vertical axis was SSC and the horizontal axis was ALDH-FITC. Cells with higher ALDH signal than the DEAB group was defined as $ALDH^{+}$ population which were in the gate of P2. After which, the $ALDH^{+}$ population was again gated by CD44 and CD24.The vertical axis was CD44-APC and the horizontal axis was CD24-PE. Cells with higher CD44 signal than the cells stained by isotype lgG were defined as $CD44^{high}$, so was the detection of CD24. The cells in Q1-1 were $CD44^{high}CD24^{low}ALDH^{+}$ cells.

Supplementary Fig 3

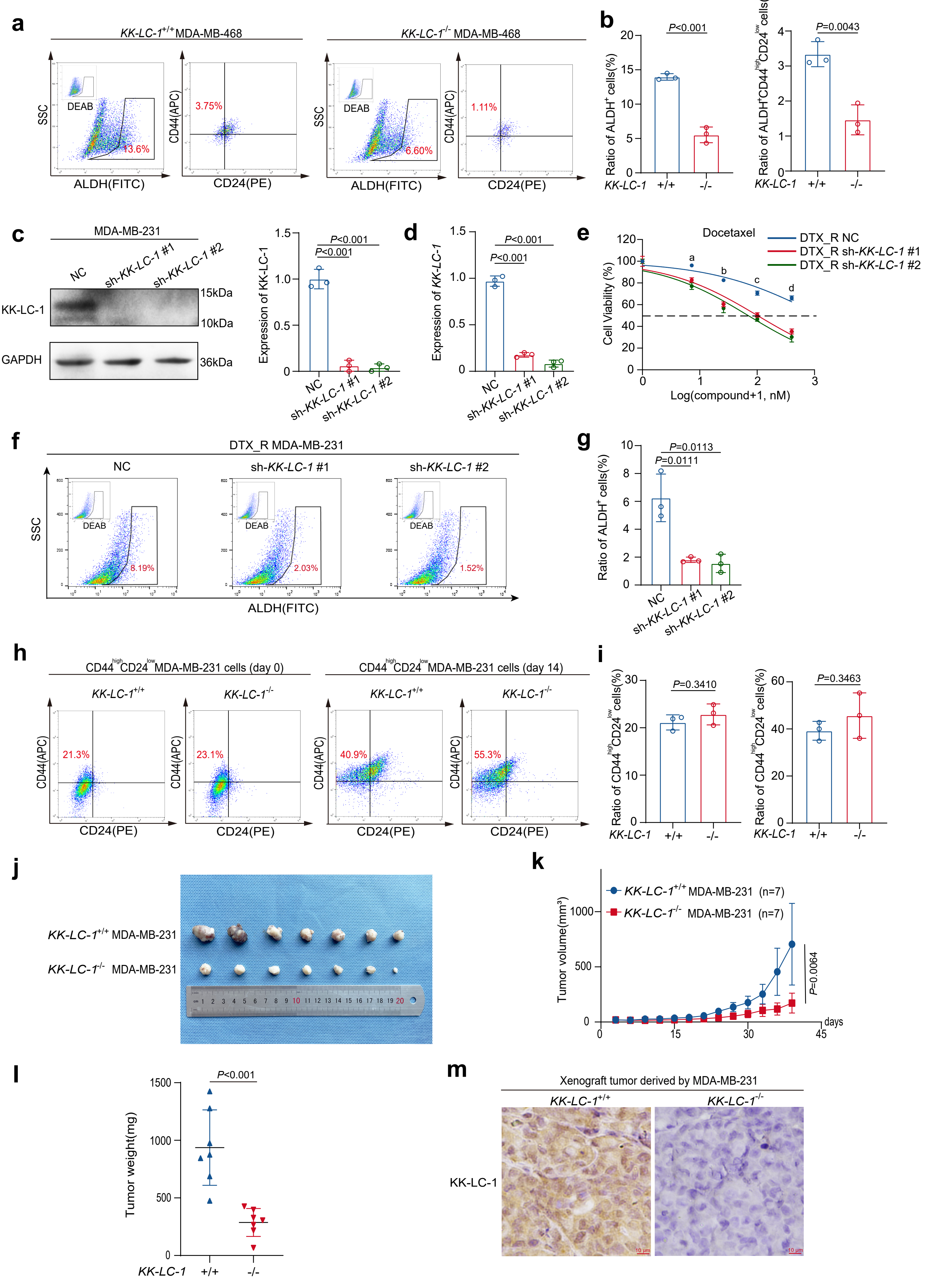

Supplementary Fig 3

KK-LC-1 is associated with drug resistance ability and $ALDH^+$ cell ratio in DTX_R cells.

a,b: Proportion of $ALDH^+$ cells and $ALDH^+CD44^{high}CD24^{low}$ cells in *KK-LC-1*$^{-/-}$ MDA-MB-468 cells and *KK-LC-1*$^{+/+}$ MDA-MB-468 cells; data are presented as mean±SD from three biologically independent experiments, statistical significance was determined using Student's t test and was two sided.

c: Lentivirus efficacy verified by Western blotting; data are presented as mean±SD from three biologically independent experiments, statistical significance was determined using Student's t test and was two sided.

d: Lentivirus efficacy verified by qRT-PCR analysis; data are presented as mean±SD from three biologically independent experiments, statistical significance was determined using Student's t test and was two sided.

e: Drug susceptibility test of docetaxel-resistant MDA-MB-231 cells transduced with sh-*KK-LC-1*#1, sh-*KK-LC-1*#2 and negative control (NC) lentivirus; data are presented as mean±SEM from three biologically independent experiments, 'a' indicates P=0.0075 when DTX_R sh-*KK-LC-1*#1 compared to DTX_R NC and P=0.0015 when DTX_R sh-*KK-LC-1*#2 compared to DTX_R NC treated by 6.25 nM docetaxel, 'b' indicates P=0.0014 when DTX_R sh-*KK-LC-1*#1 compared to DTX_R NC and P<0.001 when DTX_R sh-*KK-LC-1*#2 compared to DTX_R NC treated by 25 nM docetaxel, 'c' indicates P=0.0002 when DTX_R sh-*KK-LC-1*#1 compared to DTX_R NC and P<0.001 when DTX_R sh-*KK-LC-1*#2 compared to DTX_R NC treated by 100 nM docetaxel, 'd' indicates P<0.001 when DTX_R sh-*KK-LC-1*#1 compared to DTX_R NC and P<0.001 when DTX_R sh-*KK-LC-1*#2 compared to DTX_R NC treated by 400 nM docetaxel, statistical significance was determined using Student's t test and was two sided.

f,g: Proportion of $ALDH^+$ cells in docetaxel-resistant MDA-MB-231 cells transduced with sh-*KK-LC-1*#1, sh-*KK-LC-1*#2 and negative control (NC) lentivirus; data are presented as mean±SD from three biologically independent experiments, statistical significance was determined using Student's t test and was two sided.

h,i: Proportion of $CD44^{high}CD24^{low}$ cells in *KK-LC-1*$^{-/-}$ MDA-MB-231 cells and *KK-LC-1*$^{+/+}$ MDA-MB-231 cells in day 0 and day 14; data are presented as mean±SD from three biologically independent experiments, statistical significance was determined using Student's t test and was two sided.

j: Size of tumors formed by *KK-LC-1*$^{-/-}$ MDA-MB-231 cells and *KK-LC-1*$^{+/+}$ MDA-MB-231 cells in xenograft mouse models, n=7 mice.

k: Volume of tumors formed by *KK-LC-1*$^{-/-}$ MDA-MB-231 cells and *KK-LC-1*$^{+/+}$ MDA-MB-231 cells in xenograft mouse models; data are presented as mean±SD, n=7 mice for each group, statistical significance was determined using Student's t test and was two sided.

l: Weight of tumors formed by *KK-LC-1*$^{-/-}$ MDA-MB-231 cells and *KK-LC-1*$^{+/+}$ MDA-MB-231 cells in xenograft mouse models; data are presented as mean±SD, n=7 mice for each group, statistical significance was determined using Student's t test and was two sided.

m: Immunohistochemistry staining showing KK-LC-1 protein expression in tumors derived from *KK-LC-1*$^{-/-}$ MDA-MB-231 cells and *KK-LC-1*$^{+/+}$ MDA-MB-231 cells in xenograft mouse models, red scale bars indicate 10 μm. This experiment was performed 3 times independently with similar results.

Source data are provided in the Source Data file.

Supplementary Fig 4

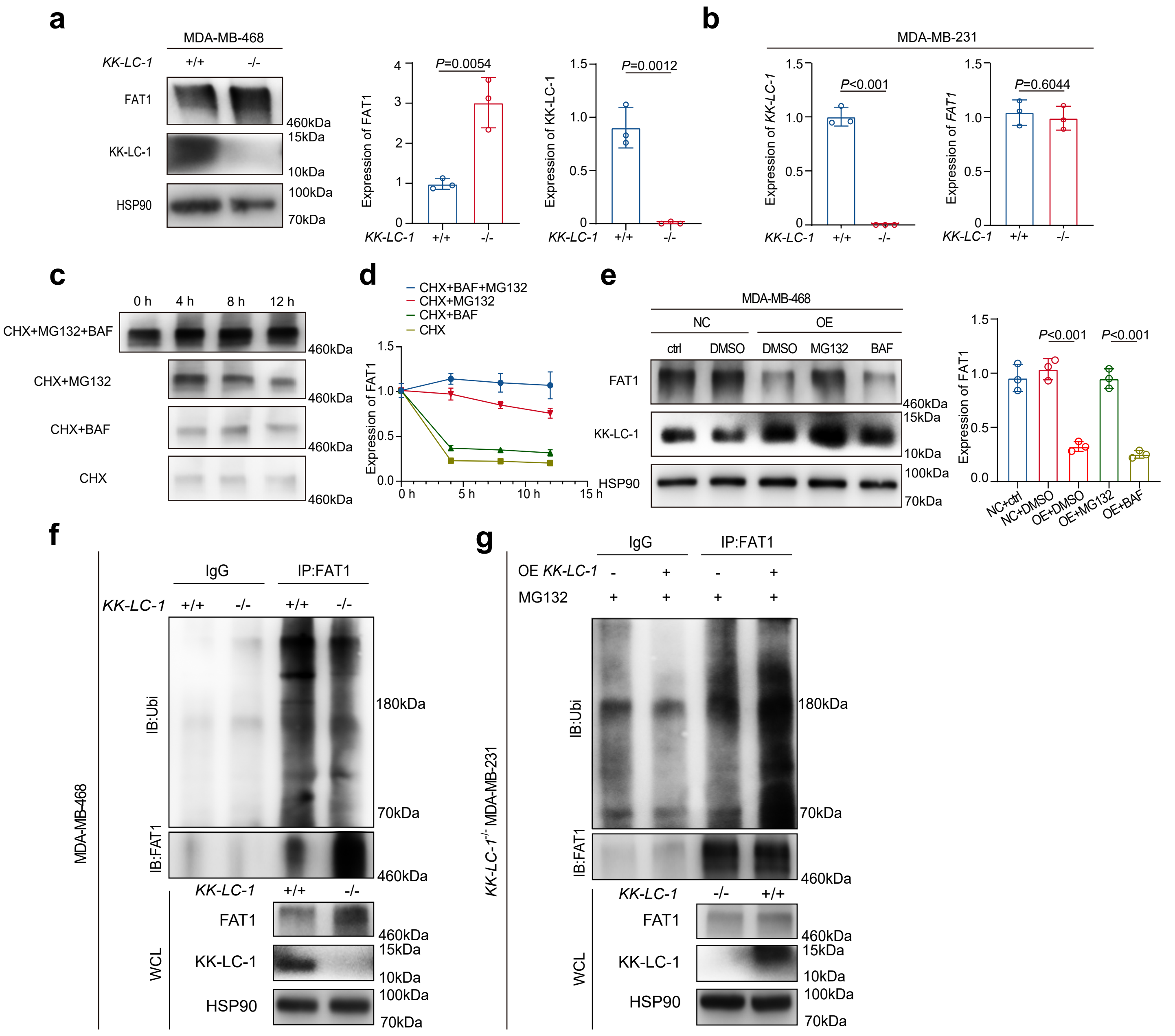

Supplementary Fig 4

KK-LC-1 regulates FAT1 at protein level and affects the ubiquitination of FAT1.

a: Western blotting showing FAT1 and KK-LC-1 protein levels in *KK-LC-1*$^{-/-}$ MDA-MB-468 cells and *KK-LC-1*$^{+/+}$ MDA-MB-468 cells; data are presented as mean±SD from three biologically independent experiments, statistical significance was determined using Student's t test and was two sided.

b: qRT-PCR showing the relative expression of *FAT1* and *KK-LC-1* mRNA in *KK-LC-1*$^{-/-}$ MDA-MB-231 cells and *KK-LC-1*$^{+/+}$ MDA-MB-231 cells; data are presented as mean±SD from three biologically independent experiments, statistical significance was determined using Student's t test and was two sided.

c,d: Western blotting showing FAT1 protein levels in MDA-MB-231 cells treated with different combinations of cycloheximide, MG132 and bafilomycin at different time points; data are presented as mean±SD from three biologically independent experiments.

e: Western blotting showing FAT1 protein levels in MDA-MB-468 cells over expressing KK-LC-1 treated with MG132 and bafilomycin; data are presented as mean±SD from three biologically independent experiments, statistical significance was determined using Student's t test and was two sided.

f: Immunoprecipitation showing ubiquitination of FAT1 protein in *KK-LC-1*$^{-/-}$ MDA-MB-468 cells and *KK-LC-1*$^{+/+}$ MDA-MB-468 cells, this experiment was performed 3 times independently with similar results.

g: Immunoprecipitation showing ubiquitination of FAT1 protein in *KK-LC-1*$^{-/-}$ MDA-MB-231 cells over expressing KK-LC-1 and NC, this experiment was performed 3 times independently with similar results.

Source data are provided in the Source Data file.

Supplementary Fig 5

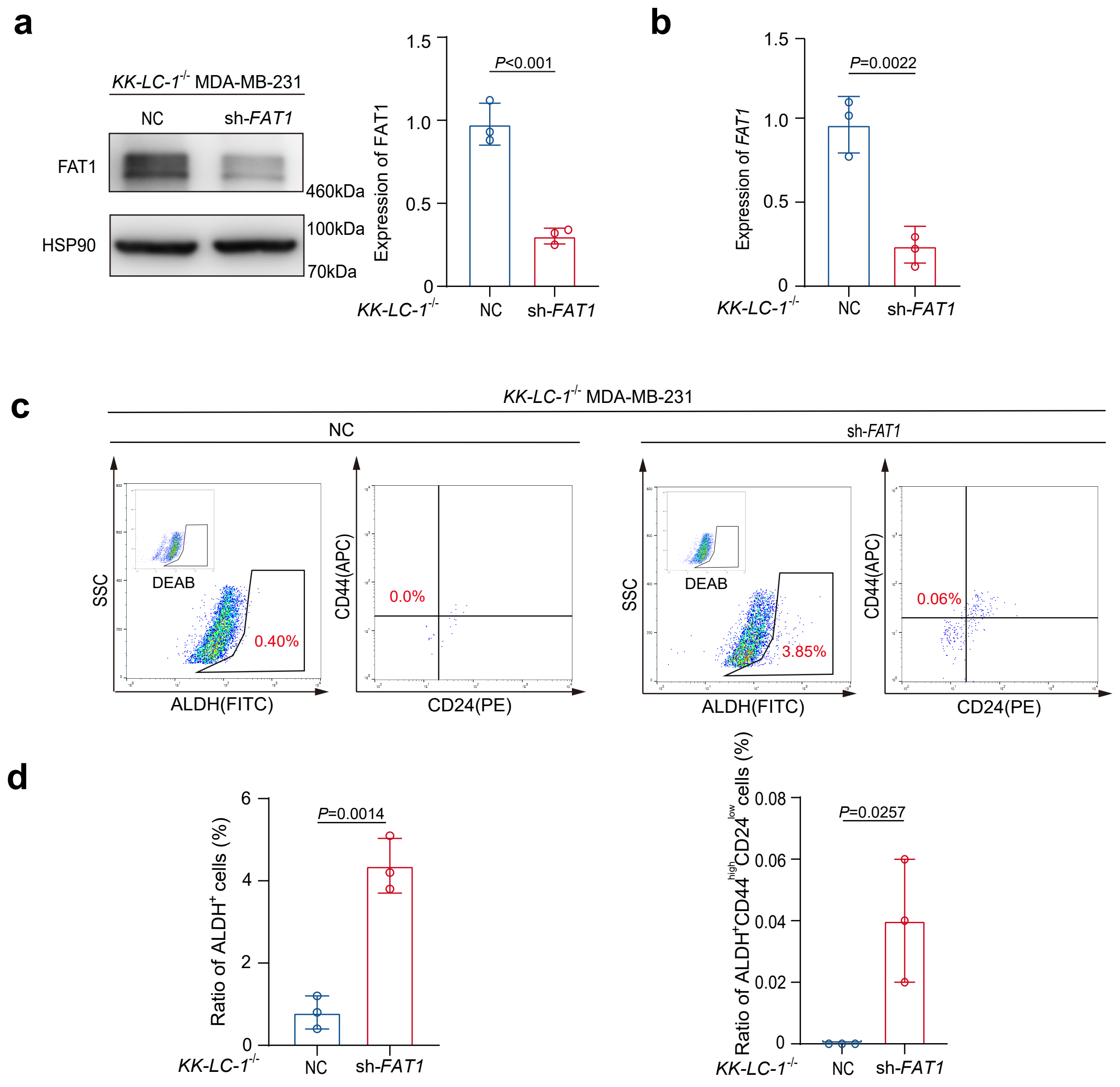

Supplementary Fig 5

Knock down of *FAT1* restores $ALDH^+$ cell ratio in *KK-LC-1*$^{-/-}$ MDA-MB-231 cells.

a: Lentivirus efficacy verified by western blot analysis; data are presented as mean±SD from three biologically independent experiments, statistical significance was determined using Student's t test and was two sided.

b: Lentivirus efficacy verified by qRT-PCR analysis; data are presented as mean±SD from three biologically independent experiments, statistical significance was determined using Student's t test and was two sided.

c,d: Proportion of $ALDH^+$ cells and $ALDH^+CD44^{high}CD24^{low}$ cells in *KK-LC-1*$^{-/-}$ MDA-MB-231 cells transfected with sh-NC and *KK-LC-1*$^{-/-}$ MDA-MB-231 cells transfected with sh-*FAT1*; data are presented as mean±SD from three biologically independent experiments, statistical significance was determined using Student's t test and was two sided.

Source data are provided in the Source Data file.

Supplementary Fig 6

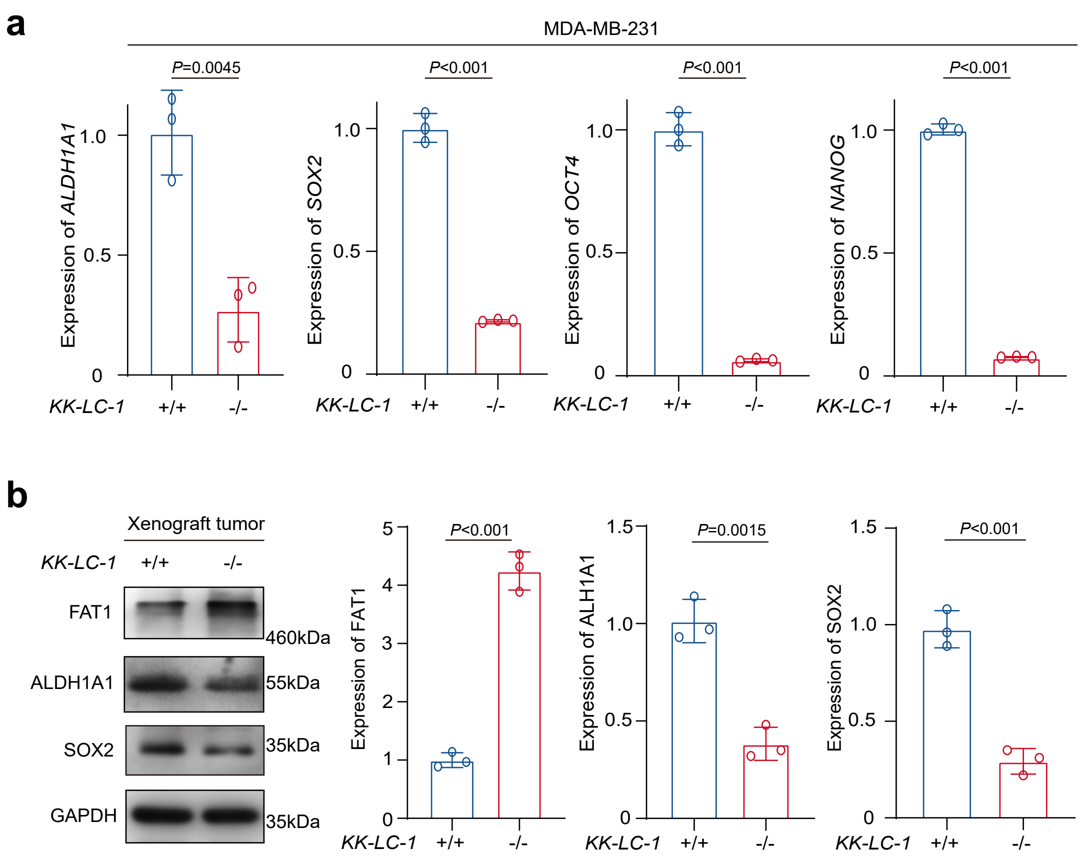

Supplementary Fig 6

KK-LC-1 regulates the expression of canonical stemness transcription factors.

a: *ALDH1A1*, *SOX2*, *OCT4* and *NANOG* mRNA levels in *KK-LC-1*$^{-/-}$ MDA-MB-231 cells and *KK-LC-1*$^{+/+}$ MDA-MB-231 cells; data are presented as mean±SD from three biologically independent experiments, statistical significance was determined using Student's t test and was two sided.

b: Western blotting showing FAT1, ALDH1A1 and SOX2 expression in xenograft tumors formed by *KK-LC-1*$^{-/-}$ MDA-MB-231 cells and *KK-LC-1*$^{+/+}$ MDA-MB-231 cells; data are presented as mean±SD from three biologically independent experiments, statistical significance was determined using Student's t test and was two sided.

Source data are provided in the Source Data file.

Supplementary Fig 7

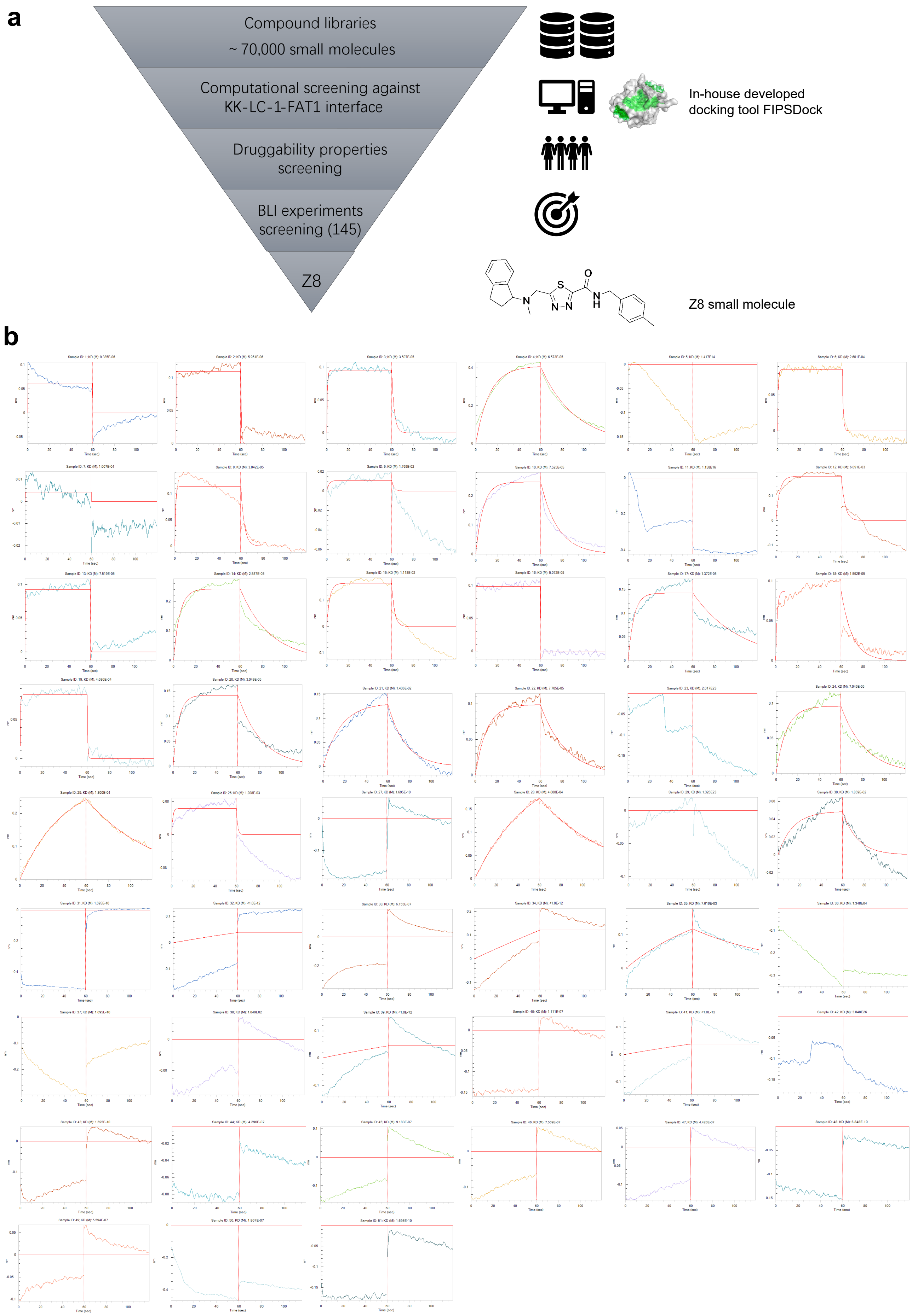

Supplementary Fig 7

Screening of small molecules which could bind with KK-LC-1.

a: Schematic depicting the selecting of small molecule of Z8.

b: Association-dissociation curves for binding of a single concentration of small molecule compounds to KK-LC-1 protein.

Supplementary Fig 8

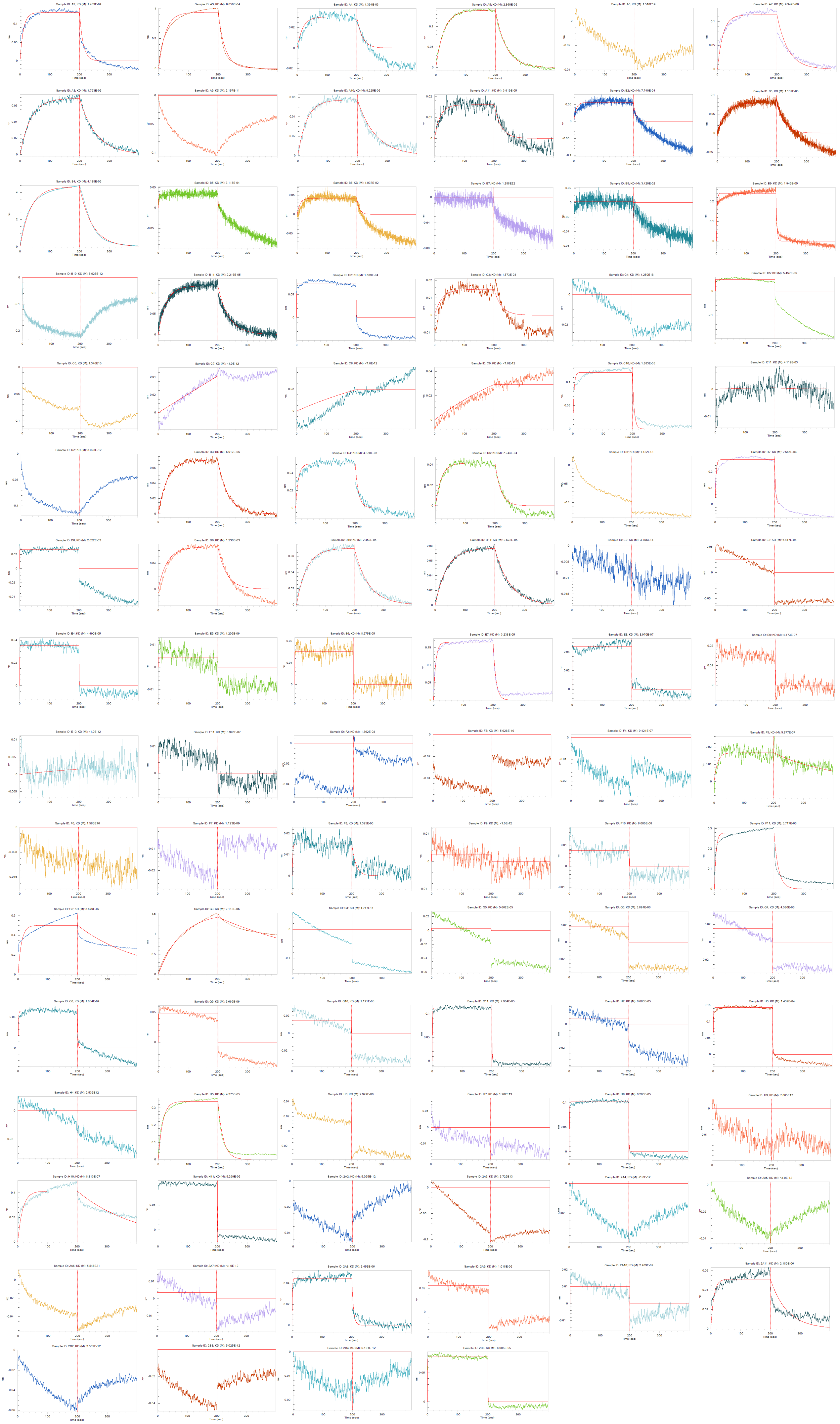

Supplementary Fig 8

Screening of small molecules which could bind with KK-LC-1.

Association-dissociation curves for binding of a single concentration of small molecule compounds to KK-LC-1 protein.

Supplementary Fig 9

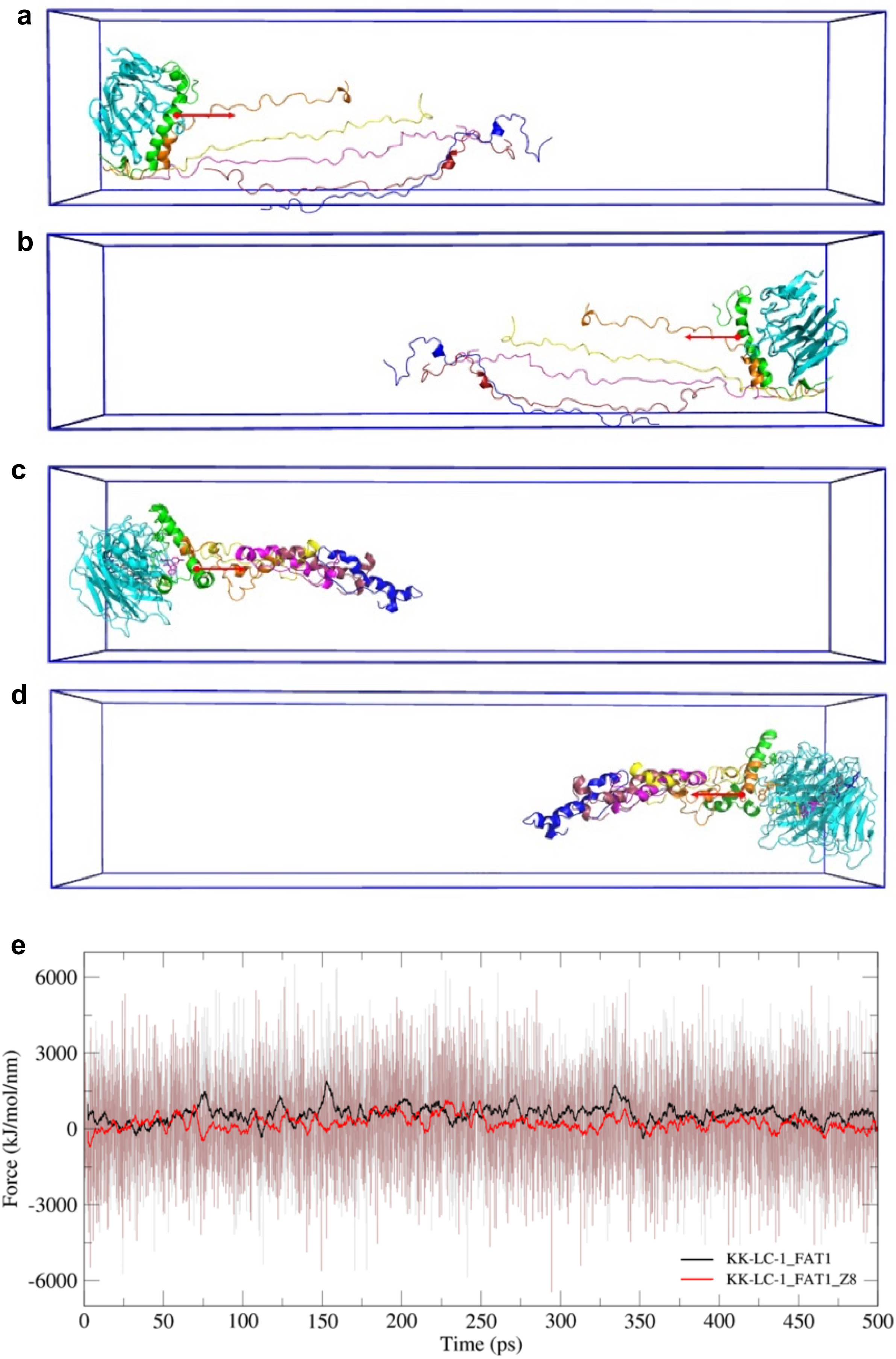

Supplementary Fig 9

Steered Molecular Dynamics (SMD) simulations on KK-LC-1-Z8-FAT1 complex.

a-b: Structure comparisons of KK-LC1-FAT1 system during the 1 ns SMD simulations.

c-d: Structure comparisons of Z839878730-KK-LC1-Laminin G triple system during the 1 ns SMD simulations. Cyan cartoon structure represents FAT1 conformations. Green, orange, yellow, magenta, raspberry and blue cartoon structure represent KK-LC-1 for the conformation at 0 ps, 200 ps, 400ps, 600 ps, 800ps and 1000ps.

e: Time dependence of rupture forces between the KK-LC-1 and FAT1 during SMD simulations.

Supplementary Fig 10

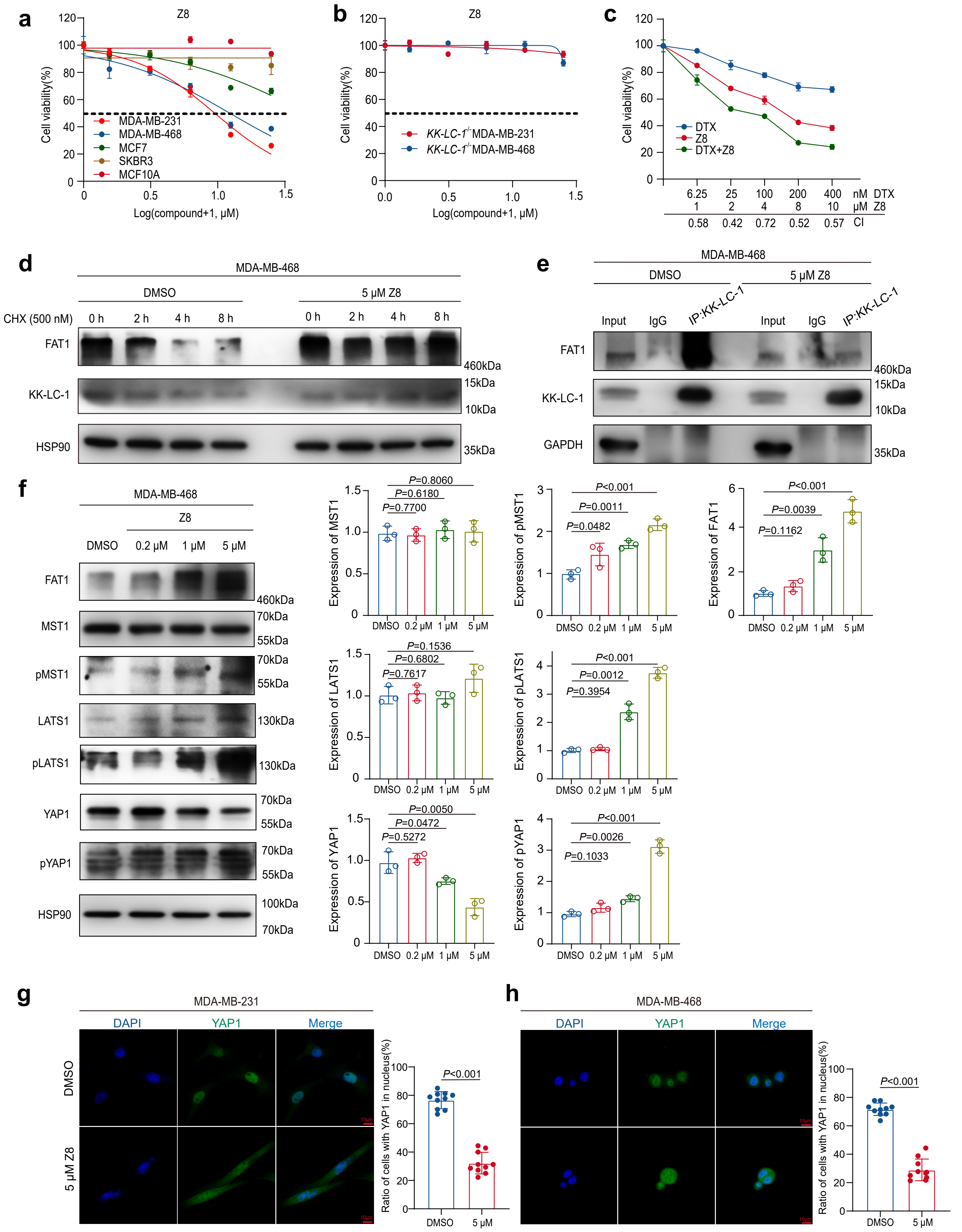

Supplementary Fig 10

Z839878730 (Z8) disrupted KK-LC-1-FAT1 protein-protein interactions and prevented FAT1 degradation thus activated Hippo signaling pathway.

a: Drug susceptibility test of Z8 in different cell lines; data are presented as mean±SEM from three biologically independent experiments, with IC_{50} as follows: 9.5 (7.52-12.23, 95% CI) μM for MDA-MB-231, 12.31 (7.86-24.04, 95% CI) μM for MDA-MB-468 and 45.90 (26.36-193.6, 95% CI) μM for MCF7. IC_{50} was estimated using non-linear fitting method.

b: Drug susceptibility test of Z8 in *KK-LC-1*$^{-/-}$ MDA-MB-231 cells and *KK-LC-1*$^{-/-}$ MDA-MB-468 cells; data are presented as mean±SEM from three biologically independent experiments.

c: Drug susceptibility test of Z8 combined with docetaxel on docetaxel-resistant MDA-MB-231 cells; data are presented as mean±SEM from three biologically independent experiments and CI<1.0 means synergistic effect which was calculated by Compusyn software.

d: Degradation of FAT1 was reduced by treatment of MDA-MB-468 cells with 5 μM Z8 compared to DMSO, this experiment was performed 3 times independently with similar results.

e: Co-immunoprecipitation of FAT1 and KK-LC-1 was reduced by treatment of MDA-MB-468 cells with 5 μM Z8 compared to DMSO, this experiment was performed 3 times independently with similar results.

f: Western blotting showing FAT1, MST1, pMST1, LATS1, pLATS1, YAP1, and pYAP1 protein levels in MDA-MB-468 cells treated with different concentrations of Z8 or DMSO; data are presented as mean±SD from three biologically independent experiments, statistical significance was determined using Student's t test and was two sided.

g,h: Immunofluorescence showing the distribution of YAP1 protein in MDA-MB-231/468 cells when treated with DMSO or 5 μM Z8, red scale bars indicate 10μm; data are presented as mean±SD from 10 randomized views, this experiment was performed 3 times independently with similar results, statistical significance was determined using

Student’s t test and was two sided.

Source data are provided in the Source Data file.

Supplementary Fig 11

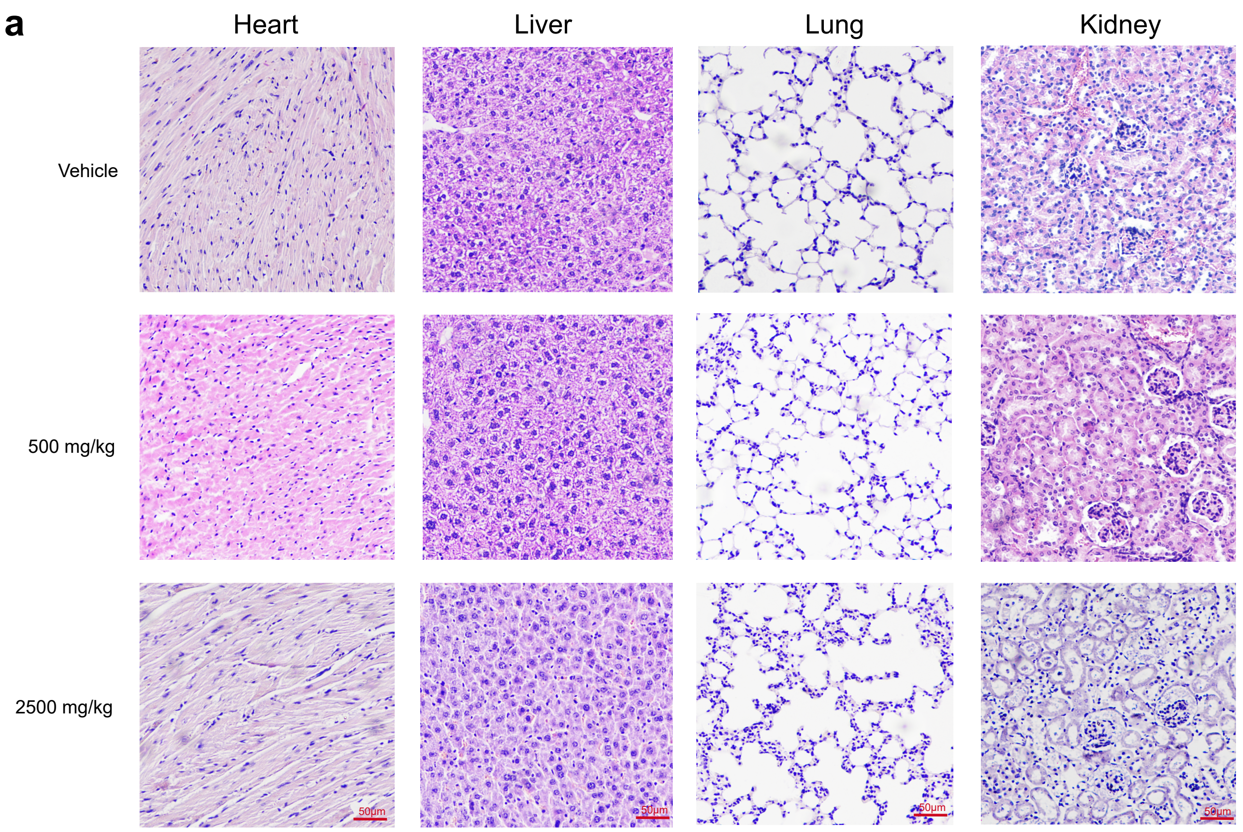

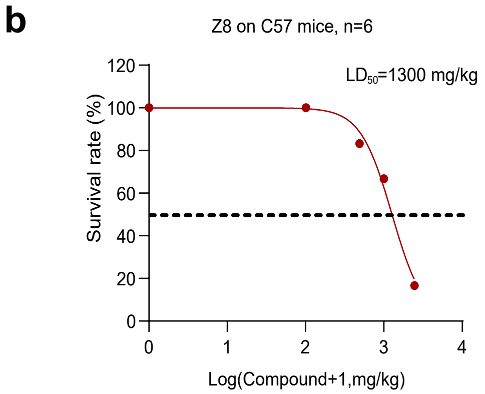

Supplementary Fig 11

Small molecule compound Z8 displays favourable safety profile.

a: H&E staining of tissues from mice treated with different concentrations of Z8 in an acute toxicity test, this experiment was performed 3 times independently with similar results.

b: LD_{50} of Z8 tested on C57 mice, n=6 mice for each group, LD_{50} was estimated using non-linear fitting method.

Source data are provided in the Source Data file.

Supplementary Table 1

Statistical results of limiting dilution assay using ALDH^{+} MDA-MB-231 cells and ALDH^{-} MDA-MB-231 cells upon *KK-LC-1* knockdown. (Statistical significance was determined using t-statistic approach and was one sided)

	ALDH^{+} cells			ALDH^{-} cells		
Cell number	NC	sh-*KK-LC-1*#1	sh-*KK-LC-1*#2	NC	sh-*KK-LC-1*#1	sh-*KK-LC-1*#2
1000 cells	24/24	17/24	16/24	2/24	2/24	2/24
100 cells	52/60	32/60	35/60	6/60	7/60	5/60
10 cells	34/60	11/60	13/60	1/60	0/60	1/60
Sphere formation frequency	1/31 (1/28-1/41)	1/267 (1/185-1/383)	1/327 (1/227-1/472)	1/3657 (1/1787-1/7484)	1/3244 (1/1645-1/6400)	1/3662 (1/1789-1/7499)
P-value		*P*<0.001	*P*<0.001		*P*=0.805	*P*=0.998

Supplementary Table 2

Statistical results of limiting dilution assay using $ALDH^+$ MDA-MB-231 cells upon *KK-LC-1* knockdown. (Statistical significance was determined using t-statistic approach and was one sided)

Cell number	sh-NC	sh-*KK-LC-1*#1
2×10^4 cells	6/6	3/6
5×10^3 cells	5/6	2/6
2×10^3 cells	4/6	1/6
500 cells	4/6	0/6
Tumor initiating frequency (TIC)	1/1426 (1/733-1/2774)	1/20878 (1/9015-1/48354)
P-value		$P < 0.001$

Supplementary Table 3

Statistical results of limiting dilution assay using $ALDH^+$ MDA-MB-231 cells and $ALDH^-$ MDA-MB-231 cells upon cultured with small molecule compound Z8. (Statistical significance was determined using t-statistic approach and was one sided)

	$ALDH^+$ cells		$ALDH^-$ cells	
Cell number	DMSO	5 μM Z8	NC	5 μM Z8
1000 cells	23/24	16/24	2/24	2/24
100 cells	50/60	34/60	5/60	6/60
10 cells	30/60	12/60	1/60	0/60
Sphere formation frequency	1/52 (1/40-1/70)	1/269 (1/187-1/387)	1/3663 (1/1789-1/7500)	1/3657 (1/1787-1/7484)
P-value		*P*<0.001		*P*=0.998

Supplementary Table 4

Demographic characteristics of patients with TNBC stratified by KK-LC-1 expression level. (Statistical significance was determined using chi-squared test and was one sided)

Clinical features	KK-LC-1 expression (Grade 1, 2)（%）	KK-LC-1 expression (Grade 3, 4)（%）	*P*-value
Patient number	87（60.4）	57（39.6）	-
Age			>0.999
≤51	48（55.2）	31（54.3）	
>51	39（44.8）	26（45.6）	
T stage			0.0009
1	49（56.3）	21（36.8）	
2	32（36.8）	19（33.3）	
3	6 （6.9）	17（29.8）	
N stage			<0.0001
0	45（51.7）	8 （14.0）	
1	23（26.4）	19（33.3）	
2	14（16.1）	18（31.6）	
3	5 （5.8）	12（21.1）	
Menopausal status			0.5685
Yes	34（39.1）	25（43.9）	
No	53（60.9）	32（56.1）	

Supplementary Table 5

Antibody information

Antibody	Dilution	Application	Cat number	Manufacturer
KK-LC-1	1:600	WB, IHC	25708-1-AP	Proteintech
KK-LC-1	1:200	IP	KKLC1-121AP	FabGennix
FAT1	1:200/1:1000	IP, WB	NBP2-32274	Novus
FAT1	1:1000	IHC	Ab198892	Abcam
ALDH1A1	1:500	IHC, IF, WB	15910-1-AP	Proteintech
SOX2	1:1000	WB, IF	20118-1-AP	Proteintech
OCT4	1:1000	WB	11263-1-AP	Proteintech
NANOG	1:1000	WB	14295-1-AP	Proteintech
MST1	1:1000	WB	22245-1-AP	Proteintech
pMST1	1:1000	WB	80093-1-RR	Proteintech
LATS1	1:1000	WB	17049-1-AP	Proteintech
pLATS1	1:1000	WB	AP0904	Abclonal
YAP1	1:1000	WB, IF, IHC	13584-1-AP	Proteintech
pYAP1	1:1000	WB	AP0489	Abclonal
GAPDH	1:5000	WB	10494-1-AP	Proteintech
HSP90	1:5000	WB	60318-1-Ig	Proteintech
Alexa Fluor 488-(green) conjugated secondary	1:400	IF	ab150077	Abcam
Alexa Fluor 594-(red) conjugated secondary	1:400	IF	ab150080	Abcam
CD44	1:500	FC	12211-MM02-A	SinoBiological
CD24	1:100	FC	130-112-656	Miltenyi

Supplementary Table 6

Primer sequence for qRT-PCR

Gene	Forward sequence	Reverse sequence
KK-LC-1	GAGCAGCATTCTGTGTGCCT	CCCGAGAGAGGTCGTAGACTG
MMP1	AAAATTACACGCCAGATTTGCC	GGTGTGACATTACTCCAGAGTTG
FAT1	CCTTCCAACAGCCACATCCACTAC	TTGAACCGTGAGCGTGTAACCTG
SOX2	GTATCAGGAGTTGTCAAGGCAGAG	TCCTAGTCTTAAAGAGGCAGCAAAC
OCT4	GTGGAGGAAGCTGACAACAA	ATTCTCCAGGTTGCCTCTCA
NANOG	CCTGTGATTTGTGGGCCTG	GACAGTCTCCGTGTGAGGCAT
ALDH1A1	GCACGCCAGACTTACCTGTC	CCTCCTCAGTTGCAGGATTAAAG
GAPDH	GGAGCGAGATCCCTCCAAAAT	GGCTGTTGTCATACTTCTCATGG
