# Supplementary material for: KK-LC-1 as a therapeutic target to eliminate ALDH+ stem cells in triple negative breast cancer
Source: Nat Commun. 2023 May 5;14:2602. doi: 10.1038/s41467-023-38097-1 (PMC10163259; doi:10.1038/s41467-023-38097-1)
Supplement: Supplementary file 3 — Supplementary Dataset 1 [file 41467_2023_38097_MOESM3_ESM.zip › Supplementary Data 1/Description of Additional Supplementary Files.docx]

Description of Additional Supplementary Files

**Supplementary Data 1** contains two tables, one with a list of up-regulated genes in docetaxel-resistant MDA-MB-231 compared to wild control 231 cells (231NC vs. 231Doc deg list), and the other with a list of up-regulated genes in triple-negative breast cancer tissue compared to normal tissue in the TCGA dataset (TNBC vs. normal deg list). The up-regulated genes are those with Fold Change greater than 1.5 and P-value less than 0.
