# Supplementary material for: KK-LC-1 as a therapeutic target to eliminate ALDH+ stem cells in triple negative breast cancer
Source: Nat Commun. 2023 May 5;14:2602. doi: 10.1038/s41467-023-38097-1 (PMC10163259; doi:10.1038/s41467-023-38097-1)
Supplement: Supplementary file 4 — Supplementary Dataset 2 [file 41467_2023_38097_MOESM4_ESM.zip › Supplementary Data 2/Description of Additional Supplementary Files.docx]

Description of Additional Supplementary Files

**Supplementary Data 2** contain two tables, one with a list of known proteins in Hippo pathway, and the other with a list of proteins that can be immunoprecipitated with KK-LC-1 and quantified using mass spectrometry.
