# Supplementary material for: KK-LC-1 as a therapeutic target to eliminate ALDH+ stem cells in triple negative breast cancer
Source: Nat Commun. 2023 May 5;14:2602. doi: 10.1038/s41467-023-38097-1 (PMC10163259; doi:10.1038/s41467-023-38097-1)

Raw gels for Figure 5

Figure 5g

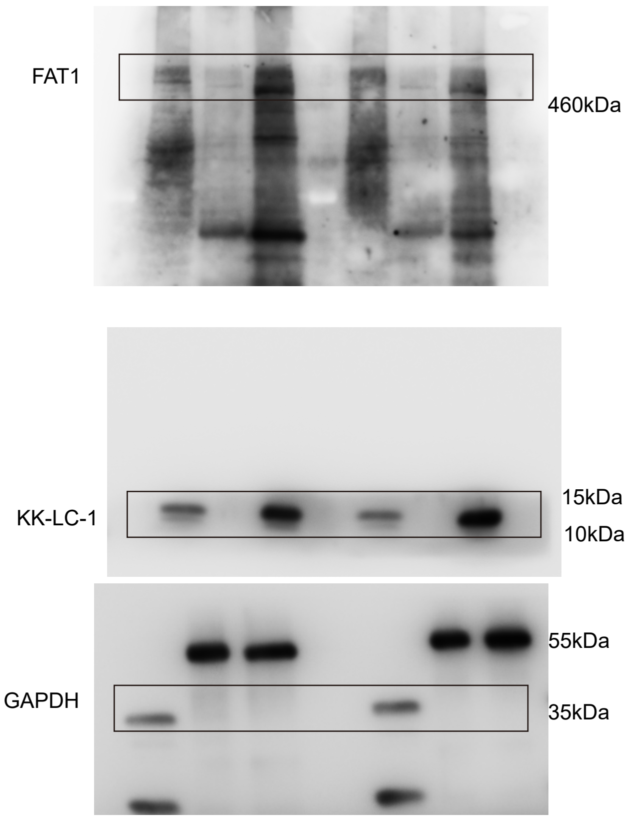

Figure 5h

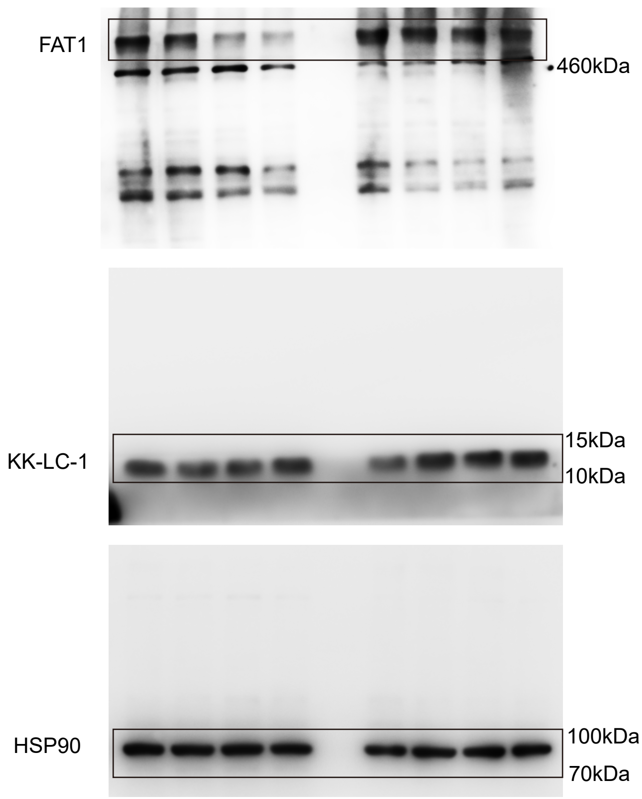

Figure 5i

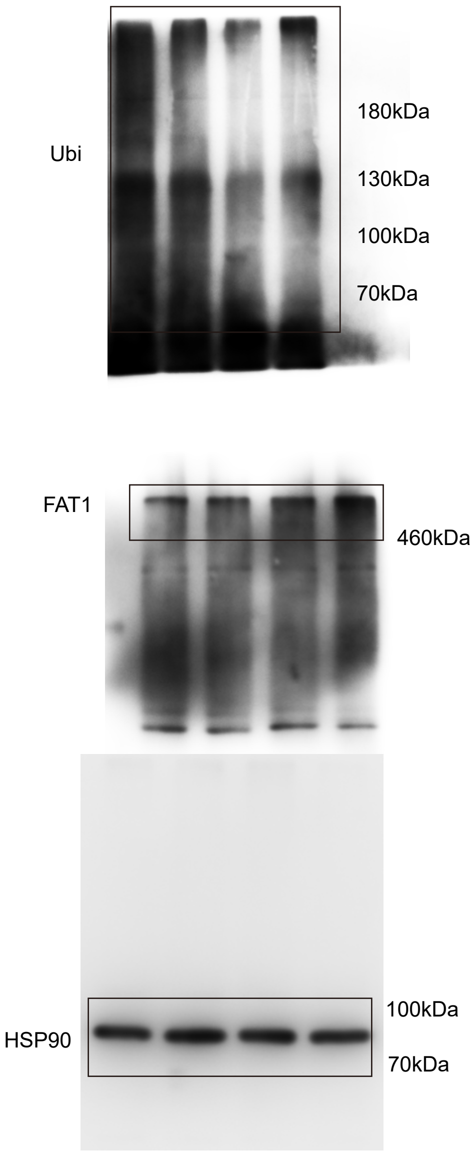

Figure 5j

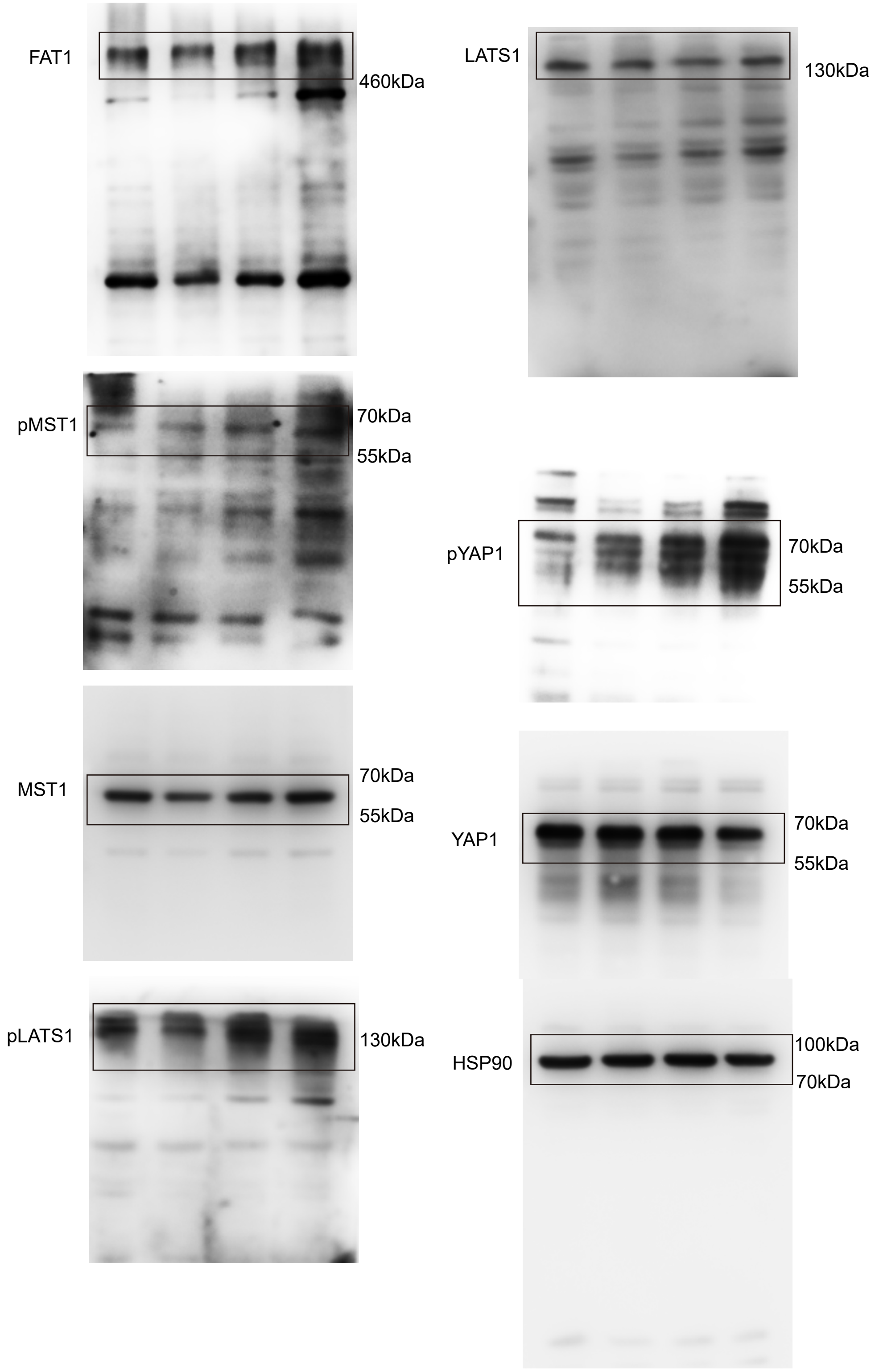

Supplement: Supplementary file 6 — Source Data file [file 41467_2023_38097_MOESM6_ESM.zip › Source Data file/Figure 5.zip!/Figure 5/Raw gels for Figure 5.pdf]
