# Supplementary material for: KK-LC-1 as a therapeutic target to eliminate ALDH+ stem cells in triple negative breast cancer
Source: Nat Commun. 2023 May 5;14:2602. doi: 10.1038/s41467-023-38097-1 (PMC10163259; doi:10.1038/s41467-023-38097-1)

Raw gels for Supplementary Figure 10

Supplementary Figure 10 d

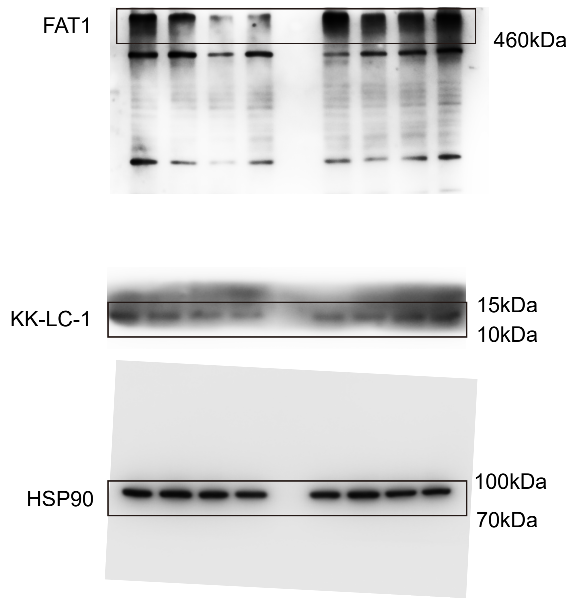

Supplementary Figure 10 e

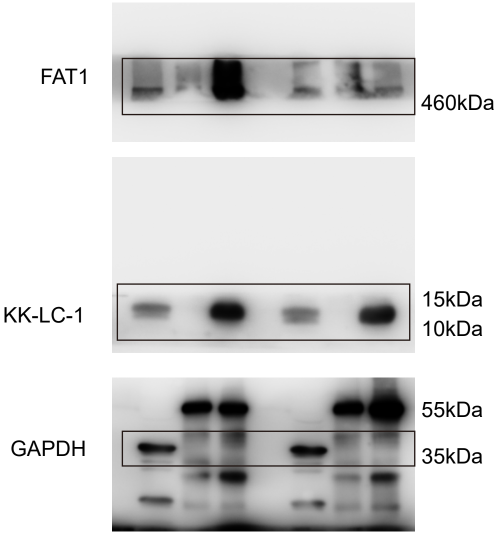

Supplementary Figure 10 f

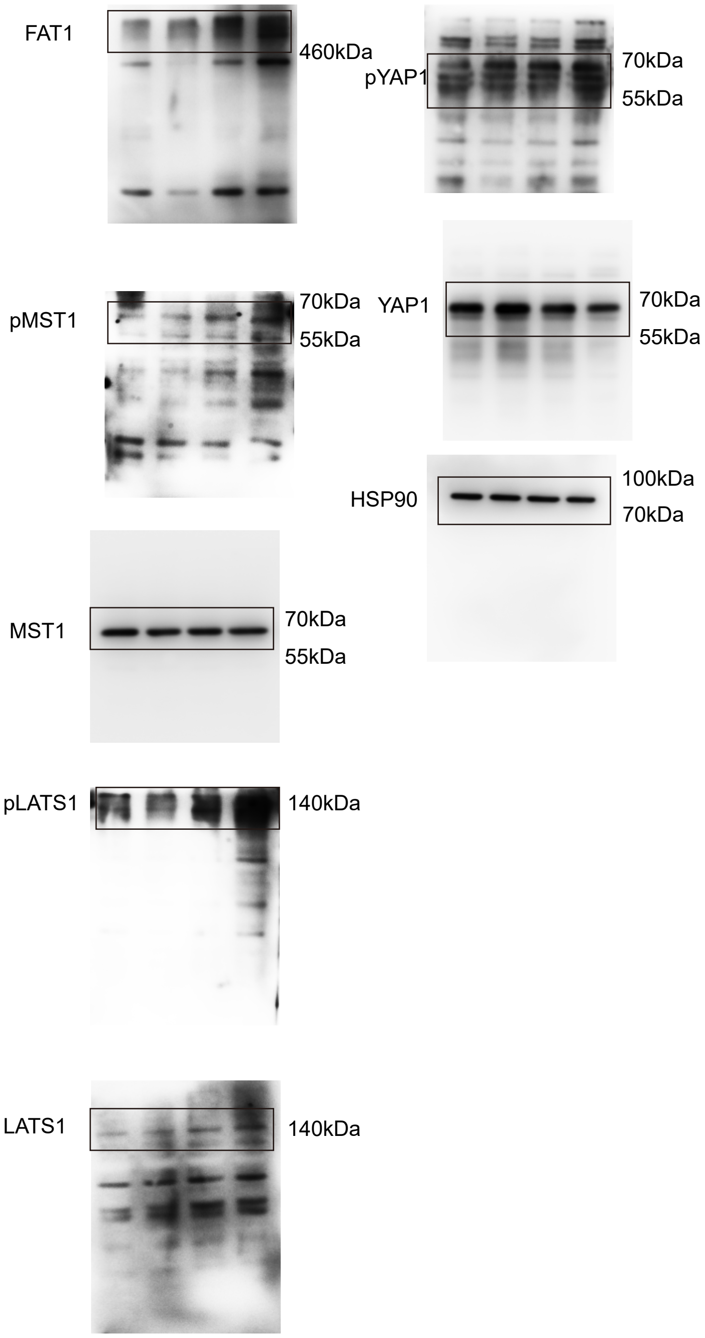

Supplement: Supplementary file 6 — Source Data file [file 41467_2023_38097_MOESM6_ESM.zip › Source Data file/Supplementary Figure 10.zip!/Supplementary Figure 10/Raw gels for Supplementary Figure 10.pdf]
