# Supplementary material for: KK-LC-1 as a therapeutic target to eliminate ALDH+ stem cells in triple negative breast cancer
Source: Nat Commun. 2023 May 5;14:2602. doi: 10.1038/s41467-023-38097-1 (PMC10163259; doi:10.1038/s41467-023-38097-1)
Supplement: Supplementary file 6 — Source Data file [file 41467_2023_38097_MOESM6_ESM.zip › Source Data file/Supplementary Figure 6.zip!/Supplementary Figure 6/Raw gels for Supplementary Figure 6.pdf]

Raw gels for Supplementary Figure 6

Supplementary Figure 6 b

FAT1

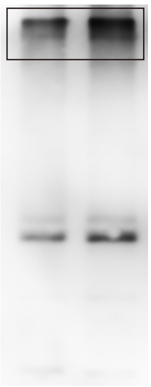

460kDa

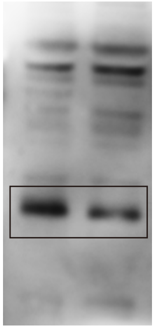

SOX2

35kDa

Supplementary Figure 6 b

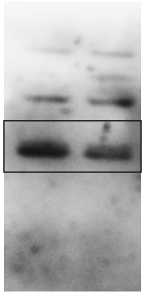

ALDH1A1

55kDa

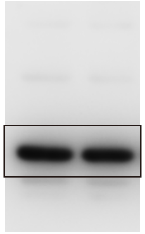

GAPDH

35kDa
